# Dialysis deserts in the Philippines: a national geospatial modeling study of dialysis services

**DOI:** 10.1080/16549716.2026.2727762

**Published:** 2026-09-07

**Authors:** Marvinson S. Fajardo, Peter M. Macharia, Grace Marie V. Ku

**Affiliations:** a Department of Public Health, Institute of Tropical Medicine, Antwerp, Belgium; b Population and Health Impact Surveillance Group, Kenya Medical Research Institute (KEMRI)-Wellcome Trust Research Programme, Nairobi, Kenya; c Department of Gerontology, Faculty of Medicine & Pharmacy, Vrije Universiteit Brussel, Brussels, Belgium; d Department of Preventive Medicine, Faculty of Medicine & Surgery, University of Santo Tomas, Manila, Philippines

**Keywords:** Quality of Care for Chronic Conditions, Noncommunicable disease, spatial analysis, hemodialysis, travel time, inequities

## Abstract

**Background:**

In the Philippines, dialysis facilities are clustered in urban centers, leaving patients with Kidney Failure (KF) in rural and geographically isolated regions severely underserved. Despite a national peritoneal dialysis (PD)-first policy since 2014, a decade of implementation has not closed this gap, and no national analysis has examined whether the existing organization of dialysis services drives geographic inequities or how integrating PD could reduce them.

**Objective:**

To assess the geographic accessibility of dialysis services and estimate potential coverage gains from integrating PD into existing hemodialysis (HD) facilities.

**Methods:**

Facility data from government registries were combined with population and terrain inputs. Geospatial analysis used least-cost path modeling with walking speeds adjusted for KF–related functional limitations.

**Results:**

In 2023, the median regional coverage within one hour of an HD facility was 67.9% (IQR: 62.4–82.6%) and within two hours of a PD facility was 51.3% (IQR: 43.2–67.6%). Coverage was lowest (36%) in Mimaropa region. Full PD integration would extend coverage to an additional 11 million people, with the largest relative gains in regions currently without any dual-modality facilities.

**Conclusions:**

Geographic inequity in KF care persists in the Philippines despite a decade of PD-first policy, exposing a gap between national financing commitments and local service reach. In settings where health insurance expansion outpaces infrastructure development, spatial inequity becomes the next frontier for reform. Integrating PD within existing HD networks offers a potential pathway to reduce travel burdens, narrow access gaps, and strengthen Universal Health Care delivery.

## Paper Context

- **Main findings:** In the Philippines, the median regional coverage was only 67.9% within one hour of a hemodialysis facility and 51.3% within two hours of a peritoneal dialysis facility, with the lowest coverage in geographically inaccessible and predominantly rural regions.
- **Added knowledge:** Integrating peritoneal dialysis into existing hemodialysis facilities would extend kidney replacement therapy to an estimated 11 million additional people, demonstrating how geospatial analysis can identify the rural populations that a home-manageable modality would reach.
- **Global health impact for policy and action:** Mapping dialysis deserts shows where health insurance expansion has not reached rural populations, and gives policymakers a basis for sequencing service integration toward the least-served regions and reducing the travel burden borne by households.

## Background

Dialysis access has emerged as a systemic health equity challenge in many low- and middle-income countries (LMICs) [1]. For people with kidney failure (KF), uninterrupted kidney replacement therapy (KRT) is essential for survival. Yet, the ability to reach dialysis services often determines if treatment can be sustained. The concept of ‘dialysis deserts’ [2,3] captures these inequities, where gaps in service distribution, financing, and delivery structurally exclude populations from care. These inequities are not only financial or clinical, but also geographic. People living in rural areas carry an added financial burden from regular transport, have more time spent commuting, and have worse outcomes [4]. A person dependent on thrice-weekly hemodialysis (HD) may spend up to 288 hours each year on travel alone, assuming a one-hour journey each way [4]. This invisible burden erodes health, strains family stability, and deepens economic insecurity [5].

In Southeast Asia, the Philippines ranks second in the burden of chronic kidney disease (CKD), measured in disability-adjusted life years (DALYs) [6], and dialysis has become the single most reimbursed service under its national health insurance (Philippine Health Insurance Corporation, or PhilHealth), surpassing maternal and surgical procedures [7]. In 2014, PhilHealth introduced the peritoneal dialysis (PD)-first policy through its Z-benefit package for catastrophic health expenditures, with the intent of incentivizing PD to become the first-line modality for KF [8]. Even with a comprehensively designed health financing package that includes PD catheter insertion, consultation, fluids, and medication, uptake has remained limited at only 4% of all KF patients [9], The sluggish growth of PD as a popular KRT option may be attributed to the separate accreditation pathway, distinct from hemodialysis, thereby creating barriers for facilities to adopt PD within their existing HD service delivery. Additionally, provision of peritoneal dialysis fluids relies on a small number of imported brands in the absence of local manufacturing, and although these consumables are financed under the PD-First Z-benefit package, they are not delivered to patients’ homes [10]. Uptake is further limited by the demands PD places on patients and caregivers, who must be trained to perform and sustain daily exchanges at home, unlike the largely passive role of the patient in facility-based hemodialysis; in Thailand, where PD has been prioritized, most patients require caregiver-assisted PD [11], underscoring the training and support burden that can discourage adoption. Most dialysis facilities are clustered in private urban hospitals and freestanding clinics, while patients in rural and archipelagic regions face the greatest barriers to access [12]. This highlights a broader phenomenon in public health: insurance expansion does not necessarily translate into improved equity for underserved populations, as those best positioned to benefit are typically located in areas with the infrastructure to absorb and capitalize on expanded funding.

Explaining these inequities requires a broader health systems perspective. The Care for Chronic Conditions Quality (CCCQ) framework emphasizes how governance, resources, financing, and service delivery interact to shape seven quality aims: safety, continuity, person-centeredness, effectiveness, accessibility, equity, and efficiency [13]. Within this lens, dialysis is a critical test of whether health systems can sustain continuous and equitable care for irreversible conditions. Levesque’s framework on access complements this by framing access as a multi-stage interaction between supply and demand, where services must be available and accommodating, and patients must be able to perceive, reach, and engage with them [14]. Within Levesque’s framework, this study operationalizes two dimensions: availability, which examines whether a dialysis facility exists within feasible travel distance, and accessibility, which asks whether the time and effort required to reach that facility are realistic for a patient with kidney failure attending treatment three times per week. Together, these frameworks establish that geographic accessibility is central to achieving equity in chronic care delivery.

Geospatial analysis provides the methodological tools to translate these frameworks into measurable insights. Spatial accessibility modeling through cost distance analysis integrates key factors that affect travel including terrain, slope, transport networks, travel speeds, land use and population distribution to estimate robust and intuitive metrics of travel-time [15–19]. This approach moves beyond simple facility counts, provider ratios or Euclidean distance to reveal whether services are realistically reachable for patients [16]. For conditions such as KF, where treatment is frequent, predictable, and survival-dependent, travel-time modeling provides a rigorous proxy for spatial accessibility.

These geospatial access methods have been extensively used and validated across other health domains. In studies conducted in Sub-Saharan Africa, Grovogui and colleagues used cost distance analysis to model geographic accessibility to facilities offering childbirth care in the Grand Conakry metropolitan area [20] while Kim and colleagues estimated travel time to hospitals offering care for breast cancer [21]. In the Philippines, geospatial approaches have been applied to surgical services and general health services [12,22]. Collectively, these studies demonstrate that spatial accessibility analysis is both feasible and policy-relevant, particularly in LMICs where terrain and infrastructure magnify inequities. Thus, studies on geographic access and how policies shape equitable service delivery must continue to be developed, especially for chronic conditions such as KF, where continuity of care can severely affect the disease course, and overall quality of life.

Despite prior applications of geospatial methods in other health domains, dialysis services in the Philippines have never been systematically examined through a spatial accessibility lens. Existing debates remain narrowly focused on financing and clinical outcomes, while geographic accessibility, which is felt directly by the patient and their relatives, has been largely overlooked. In this study, dialysis deserts are defined as geographic areas where the nearest dialysis facility lies beyond a clinically and policy-relevant time-threshold, specifically one hour for HD, and two hours for PD, making regular treatment attendance physically and financially prohibitive. This study addresses that gap by providing the first national analysis of dialysis deserts in the Philippines. The objective was to assess geographic accessibility and coverage of dialysis facilities and to estimate the potential equity gains of integrating peritoneal dialysis into existing hemodialysis networks.

## Methods

### Study setting and design

**Figure 1.**
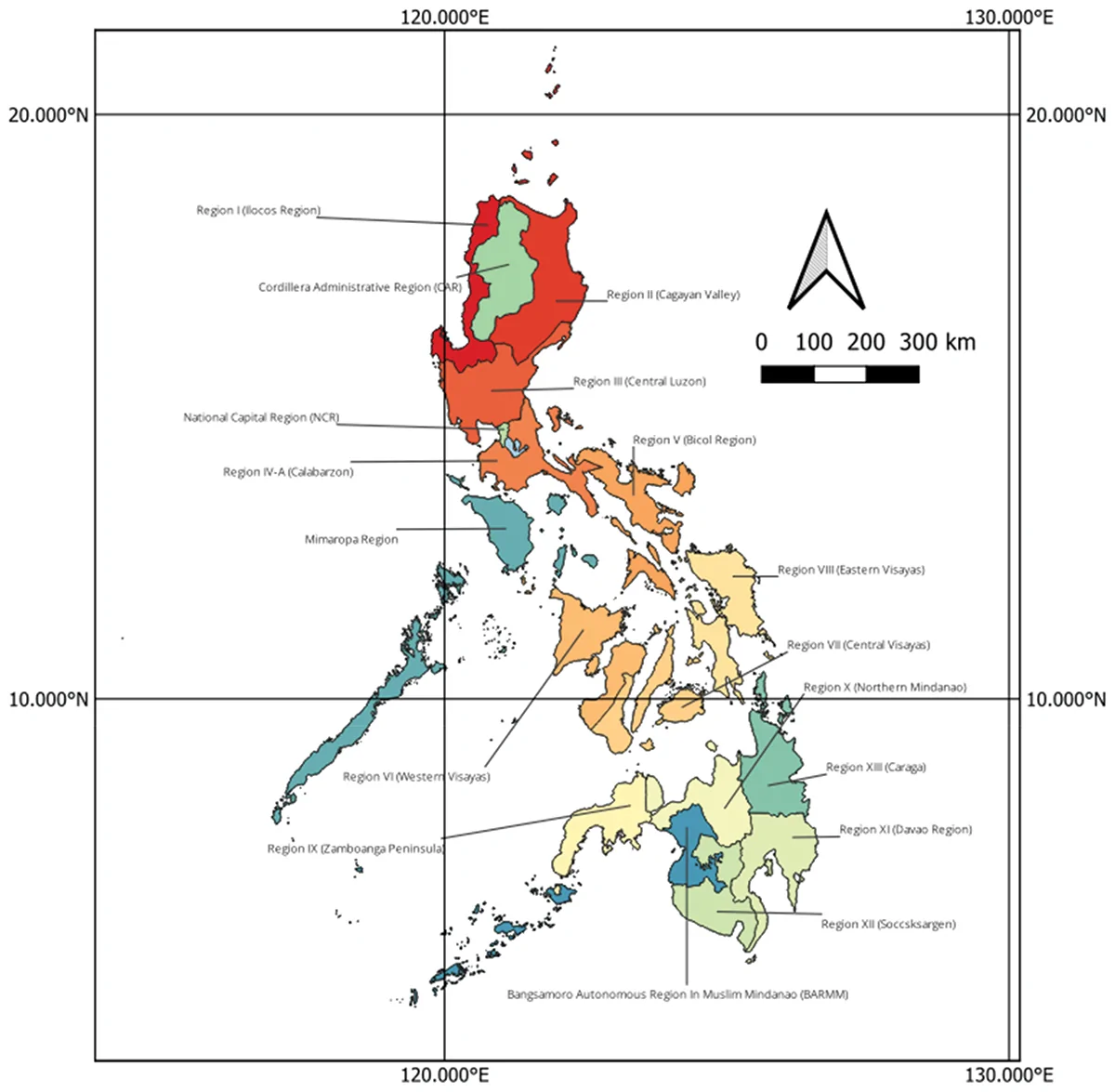
Map of the Philippines.

This national analysis covers the Philippines’ 7,641 islands organized into three island groups (Luzon- where the national capital is situated, Visayas, and Mindanao) and 18 administrative regions (Figure 1). The country’s health service delivery operates under a devolved system, with local governments responsible for managing primary care facilities and the Department of Health providing policy direction [23]. This governance arrangement, combined with geographic fragmentation, has resulted in wide variations in the implementation of health programs and investments. The Philippines was therefore an ideal setting to examine dialysis deserts and to test the potential equity impact of integrating peritoneal dialysis (PD) into hemodialysis (HD) networks.

This study employed a nationwide cross-sectional geospatial design to assess dialysis facility accessibility in the Philippines. Travel-time coverage was used as a proxy for geographic access, acknowledging that the ability to physically reach services is a prerequisite for sustaining kidney replacement therapy. This approach builds on established geospatial methods in public health and applies them to chronic disease management, where the frequency and continuity of treatment make accessibility a central equity concern [15,16].

### Data sources

#### Health facilities

All dialysis facilities accredited as of 2023 were included in the analysis. Facility data were obtained from the Department of Health Facility Registry and the PhilHealth Dialysis Facility Registry, which are separately maintained for hemodialysis and peritoneal dialysis. The retrieved registry data was manually harmonized, cleaned, and cross-validated to remove duplicates and closed sites, resulting in a total of 903 operational facilities nationwide. Because geographic coordinates were incomplete, addresses were geocoded through the Google Maps API using R and validated against administrative boundaries and OpenStreetMap reference layers. Coordinates outside the province or region were manually corrected. Facilities were classified by ownership (government or private), facility type (freestanding or hospital-based), and dialysis modality (hemodialysis only, peritoneal dialysis only, or dual). No sampling or imputation was applied.

#### Population distribution

Population data were drawn from the 2020 national census, which reported a total population of 109 million. The counts were at the region-level resolution. Gridded population data from the Philippine Statistics Authority were used to generate a 100-meter raster representing population density and distribution across the country. These rasters were aligned with regional administrative boundaries and used to compute the proportion of the population within each modeled travel-time threshold. Nationally representative sub-national data on kidney failure prevalence is not currently available in the Philippines, and the general population denominator additionally captures household-level travel burden, as patients in the Philippine context routinely attend sessions accompanied by family members.

#### Ancillary spatial data

Detailed data assembly steps are described in the Annex. Briefly, spatial datasets representing travel-time determinants included a digital elevation model (DEM), land cover, hydrography, and the road network. The DEM was sourced from the AW3D30 (JAXA) dataset, resampled to 100-meter resolution to adjust slope-dependent walking speeds. Land cover data were obtained from the 2018 Philippine GIS Data Clearinghouse and reclassified into six categories (built-up, cropland, forest, grassland, bare, and water surfaces) which represent off-road movement between transport networks. Hydrographic features, derived from the HDX ‘Philippines Waterways (OSM Export, 2025)’ layer, were considered impassable except where intersected by road bridges. The 2025 OpenStreetMap road network (HDX export) was cleaned and categorized into Motorway, Primary, Secondary, Tertiary, Pedestrian, and Impassable classes to assign realistic travel speeds by transport mode. All spatial layers were reprojected to EPSG:32651 (WGS 84 / UTM Zone 51N) for integration into AccessMod, where they were merged to construct the friction surface used to model national travel time to dialysis facilities.

### Data pre-processing

To enable integration across sources, all spatial layers were harmonized to a single projection (WGS 84 / UTM Zone 51N). Raster layers were resampled to a uniform resolution of 100 m × 100 m. A more detailed section regarding data sources, processing, and reclassification tables and technical parameters is provided in Annex.

### Facility ownership and density indicators

After data processing, facilities were stratified by region and ownership. Regional differences in service distribution were first summarized using area-based and population-based facility densities. Area-based density was defined as the number of dialysis facilities per 1,000 km^2^ of land area, while population-based density represented the number of facilities per million inhabitants. Both were measured at the regional level. These complementary measures provided an overview of geographic and demographic service availability before modeling travel-time accessibility.

### Scenario design and thresholds

Four scenarios were modeled to reflect current service availability and possible policy reforms: (1) HD-only facilities, (2) PD-only facilities, (3) the current HD+PD mix, and (4) a hypothetical expansion in which all HD facilities also offered PD.

Each scenario was modeled under both walking-only and mixed-transport (hybrid) conditions. The walking scenario simulated travel by patients without access to motorized transport, applying slope-adjusted walking speeds derived from the AW3D30 DEM and land cover–based friction values. The mixed scenario combined walking segments from residences to the nearest road with motorized travel along the classified road network, representing the most probable travel pattern for patients using private or public transport. The mixed-transport scenario assumes dry-season road conditions and represents best-case travel conditions; actual travel times for patients dependent on informal transport, seasonal roads, or inter-island ferry services are likely to be longer.

Accessibility thresholds were set at one hour for HD and two hours for PD. The one-hour threshold for HD is based on existing studies that patients traveling more than one hour each way experience significantly higher mortality and lower quality of life [24]. The two-hour threshold reflects the less frequent facility visits required for PD, generally once or twice per month, and the ensuing greater tolerance for distance [25,26]. The coverage figures reported as primary results reflect the mixed-transport scenario; walking-only estimates are provided in the sensitivity analysis to characterize access for patients without motorized transport.

### Accessibility modeling and analysis

Facility ownership (public or private), type (hospital-based or freestanding), and administrative region were used as descriptive stratifiers to illustrate structural differences in service delivery. AccessMod 5 [27], developed by the World Health Organization and partners, was selected as the primary tool for modelling geographic accessibility. The software estimates travel time and corresponding geographic coverage of health facilities within predefined time thresholds by integrating slope, land type, and road network.

The road network, land cover, and water body layer were merged to create a consolidated friction layer, which simulates the difficulty of traversing different types of terrain and road types. The model was implemented using the ‘Merge Land Cover’ and ‘Accessibility’ functions within the AccessMod software. The slope derived from the DEM was used to adjust walking speeds based on Tobler’s formula, which accounts for the speed adjustment of uphill compared to downhill walking speeds. ‘Knight’s move’ setting was used to allow improved precision of modeled estimates.

Each travel time scenario was computed the cumulative least-cost route from every populated cell to the nearest dialysis facility, minimizing total travel time regardless of administrative boundaries. Land cover types such as cropland, grassland, and urban built-up areas were assigned different walking speeds, while road networks were given motorized vehicle speeds (Annex).

The study recognizes that people with KF would have slower walking speeds compared to the healthy population. To account for these disease-related mobility limitations, a walking speed of 3 km/h [28] was applied rather than the conventional 5 km/h standard assumption in most geographic accessibility modelling studies using AccessMod5, as default parameters tend to overestimate access among chronically ill populations. This speed reflects patients with kidney failure specifically and likely represents a conservative estimate that may still overestimate mobility in frail or elderly patients on dialysis. Scenarios 3 and 4 were based on prior geospatial analyses that simulated facility expansion to assess policy-relevant scale-up options [29,30]. The travel speeds and scenarios are shown in Annex.

Sensitivity analyses were undertaken to assess the robustness of travel-time estimates to different mobility and transport assumptions. Two sets of tests were performed. First, coverage estimates generated under the standard healthy adult walking speed (5 km/h) were compared with those based on a KF-adjusted speed (3 km/h) to account for reduced mobility among dialysis patients. Second, within each walking-speed scenario, motorized travel speeds were varied by ±20% across all road classes to reflect uncertainty due to terrain, traffic, and seasonal road conditions. These analyses examined how variations in both walking and motorized speeds influenced overall accessibility outcomes. Detailed parameter values and results are provided in Annex. Coverage estimates represent potential geographic access and should not be interpreted as actual utilization, which is further shaped by facility capacity, insurance accreditation boundaries, patient preferences, and transport availability on the day of treatment.

### Map visualization and quantitative analysis

For each scenario, travel-time models were generated and overlaid with the 2020 population grid to compute the share of the population within the defined thresholds. Coverage was summarized at both national and regional levels. Map preparation and visualization were conducted in QGIS, while descriptive statistics and scenario comparisons were performed in R.

## Results

### National distribution of dialysis facilities

In 2023, a total of 903 dialysis facilities were accredited nationwide. The majority (86.3%, *n* = 779) were privately owned, while only 13.7% (*n* = 124) were operated by government institutions. By facility type, 65% were freestanding dialysis clinics and 35% were hospital-based. HD is still the dominant modality, with 851 facilities (94.2%) providing HD-only services. PD-only sites were rare, totaling just 9 (1.0%) nationwide. Only 43 facilities (4.8%) were accredited for both HD and PD (Table 1).

**Table 1.** Counts of included dialysis facilities, by ownership and by service available in the Philippines in 2023.

	HD-Only	PD-Only	HD + PD	Total
Government	98	2	24	124
Private	753	7	19	779
Total	851	9	43	903

**Figure 2.**
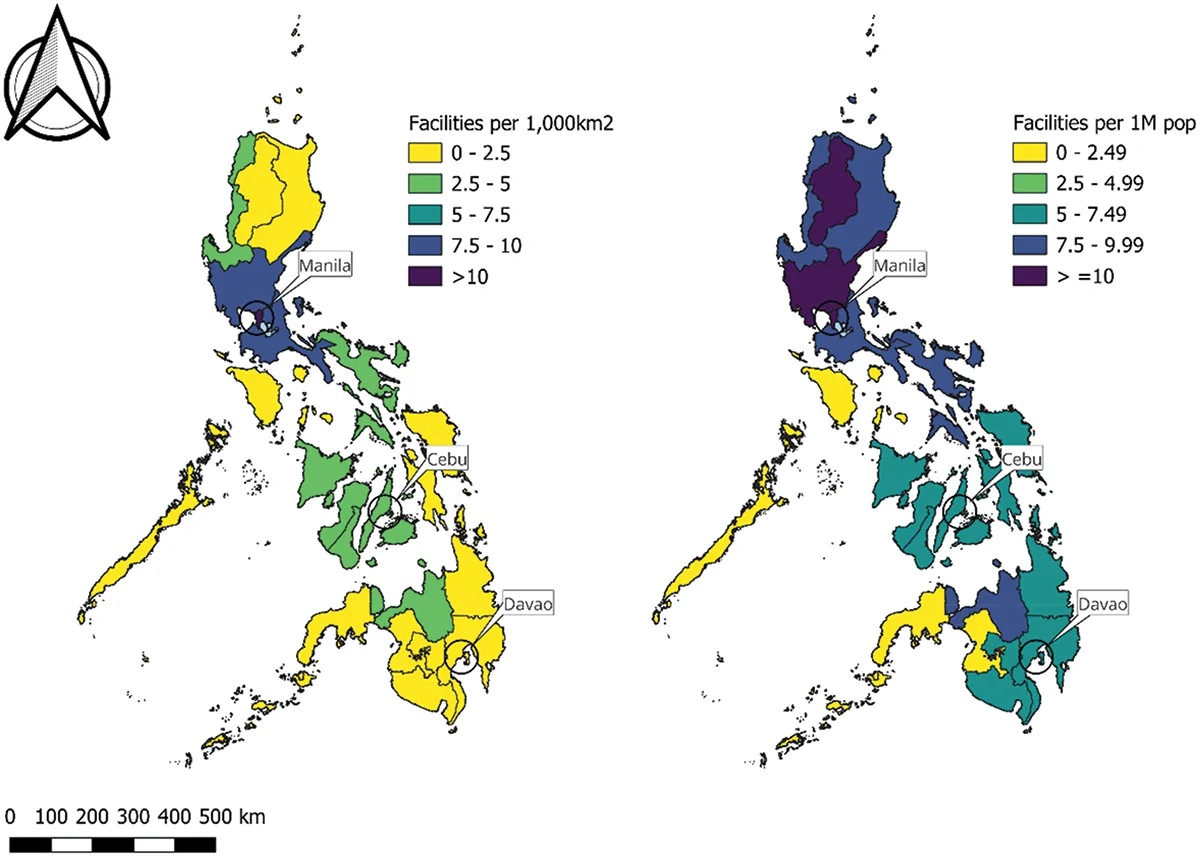
Regional dialysis facility density, by area (left), and population density (right) in Philippines, 2023.

The distribution of facilities was uneven across regions. The National Capital Region (NCR) reported the highest number, with 190 facilities (21%), corresponding to the highest area-based density (317.4 per 1,000 km^2^) and also among the highest in population-based density (14.1 per million population) (Figure 2). By contrast, the Bangsamoro Autonomous Region in Muslim Mindanao (BARMM) had only five facilities (0.5%), representing the lowest density nationally (1.01 per million). Dual-modality (HD+PD) facilities were mostly concentrated in urban Luzon, with none present in MIMAROPA (Provinces of Mindoro, Marinduque, Romblon, and Palawan), BARMM, or Soccsksargen (Provinces of South Cotabato, Cotabato, Sultan Kudarat, Sarangani, and General Santos). Among the 43 dual-modality facilities, more than half (55.8%) were government-run and in tertiary hospitals, despite the majority of overall dialysis facilities being privately owned and freestanding clinics.

These figures provide a baseline profile of dialysis infrastructure in the Philippines, showing overall facility numbers, ownership patterns, and modality availability. They form the foundation for subsequent analyses of geographic accessibility and scenario modeling.

### Sensitivity analysis on mobility assumptions

**Figure 3.**
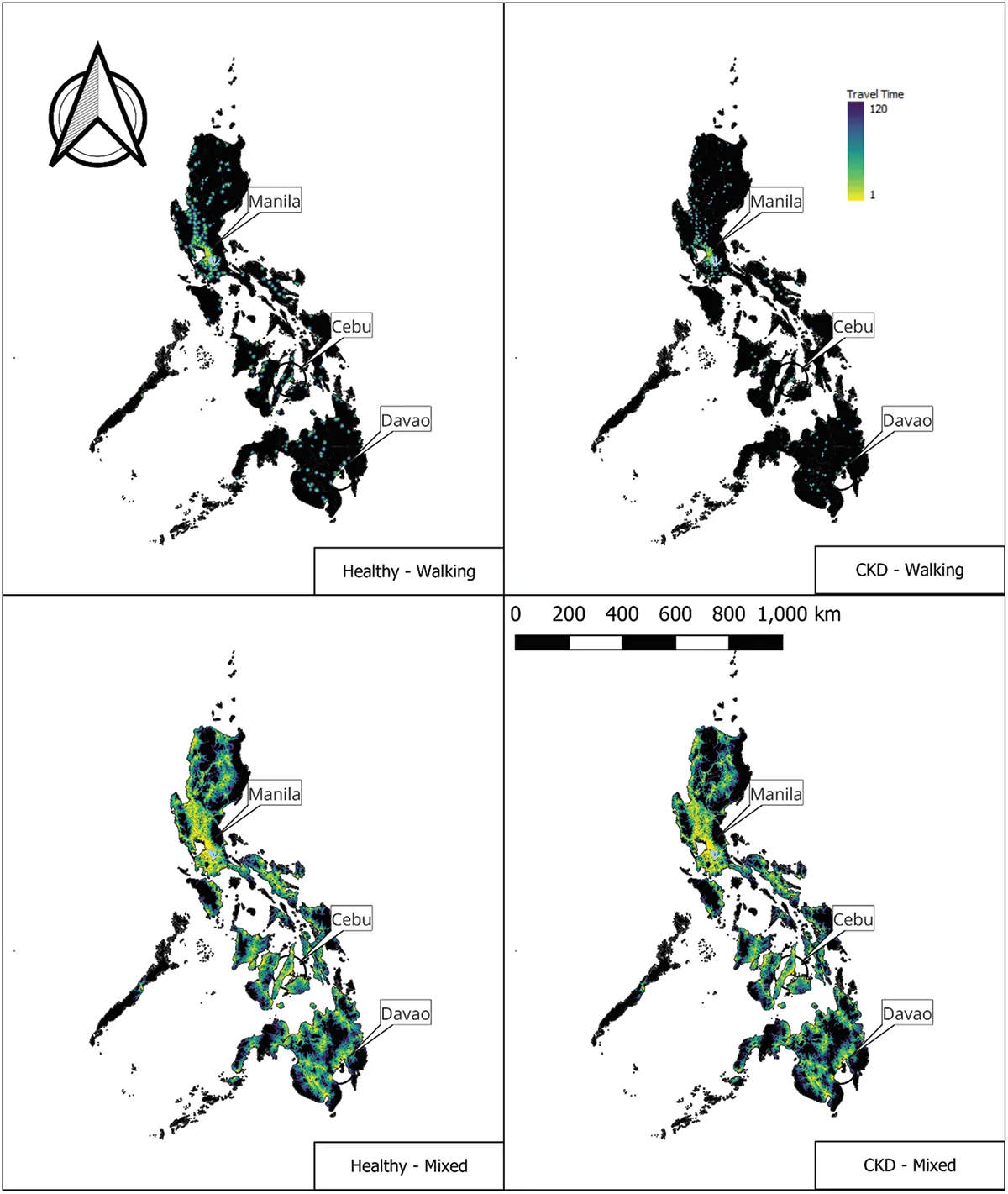
Comparing accessibility using healthy vs KF walking speeds under walking only, and mixed transport.

Accessibility estimates differed depending on whether healthy adult or KF-adjusted walking speeds were applied. Using the conventional healthy adult speed of 5 km/h, national mean coverage was 34.1% for walking only, and 74.3% for mixed-mode transport. Adjusting to 3 km/h walking speed reduced these estimates to 26.4% and 70.9% respectively. On average, the relative difference of the two travel speed assumptions was 26.3% for walking only and 5.0% for mixed-mode transportation (Figure 3).

Regional differences were consistent with the national trend, although the magnitude of overestimation varied. In regions with lower density of road networks for facility access, such as BARMM, Cagayan Valley and Eastern Visayas, the relative difference of coverage gaps between healthy and KF-adjusted assumptions exceeded 7%. In more urbanized regions where road networks predominated, differences were smaller but still present, with relative differences ranging from 1% to 2%. The largest discrepancies were observed in walking only scenarios, where slower walking speeds disproportionately reduced effective coverage, with the Zamboanga peninsula in Mindanao (42%) and Ilocos Region in Luzon (41.1%) having the highest relative differences.

Kidney failure–adjusted travel speeds are used as the reference for all subsequent scenario comparisons.

### Geographic accessibility and coverage

Modeled geographic accessibility to HD facilities exceeded that of PD facilities across all regions. The median regional coverage was 67.9% (IQR: 62.4–82.6) within one hour of an HD facility, while only 51.3% (IQR: 43.2–67.6) was within two hours of a PD facility. Coverage for PD was particularly limited in archipelagic and rural regions. In MIMAROPA, PD coverage was effectively zero, while in the Zamboanga Peninsula it fell below 25%. By contrast, the NCR and adjacent Luzon regions achieved the highest levels of coverage, exceeding 80% for HD and 70% for PD. Interestingly, MIMAROPA, which is also part of the Luzon Island Group, had the lowest population coverage for HD at 36.04%.

**Figure 4.**
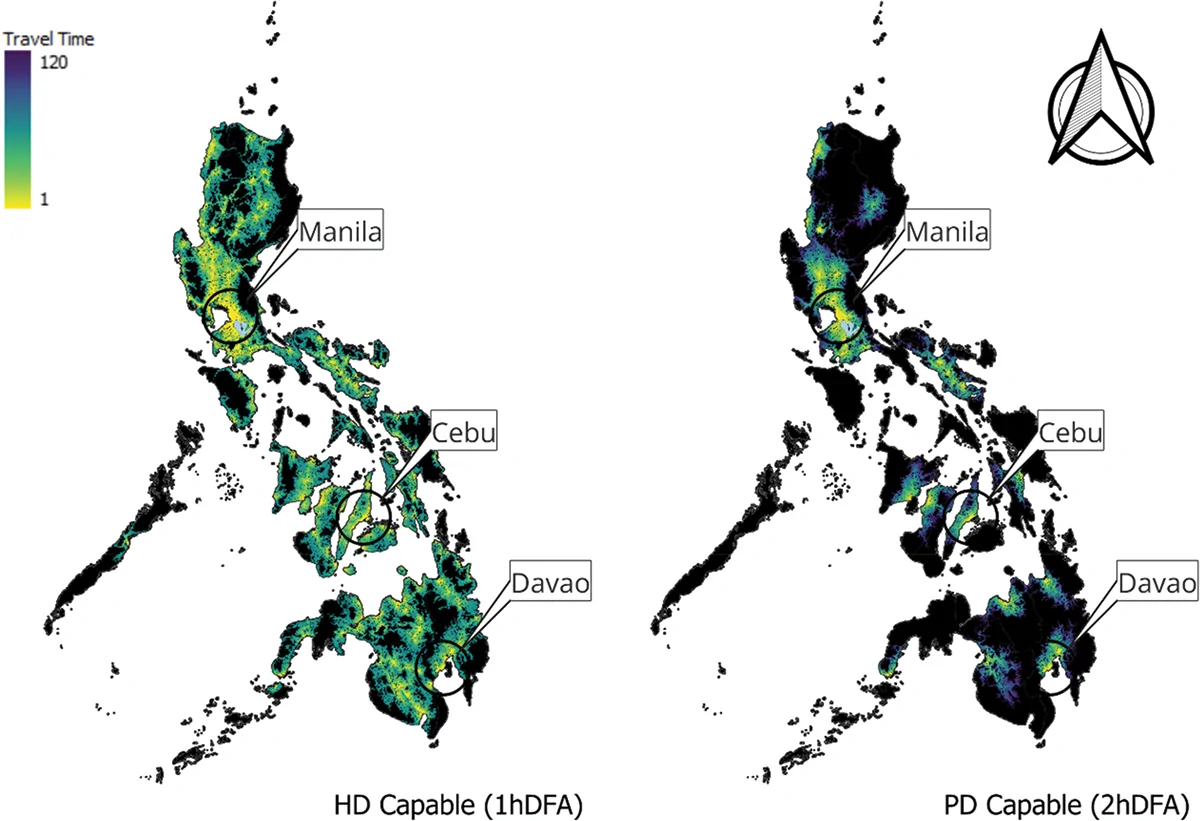
Accessibility of KRT modalities: HD (1hDFA), PD (2hDFA).

Dual-modality (HD+PD) facilities were limited in number and primarily concentrated in urban areas. Most regions had at least one dual-accredited facility, but their absence in MIMAROPA, BARMM, and Soccsksargen meant that no population in these regions could reach a site offering both modalities. Stratification by ownership showed that private facilities dominated accessibility nationally, although public facilities provided a higher share of dual-modality access. Collectively, these results indicate that under current configurations, HD remains more accessible than PD, and large geographic disparities persist in PD availability (Figure 4).

### Accessibility under integration scenarios

Simulated integration of PD into all existing HD facilities substantially increased population coverage. Compared with HD-only coverage at baseline, full integration would extend PD reach to an estimated 11.0 million people who currently have HD but do not have PD as an option (Luzon: 3.7 million, Visayas: 3.1 million, Mindanao: 4.2 million). Coverage gains were observed in all regions. The largest relative gains occurred in the least-served regions, reaching 50.3% in Zamboanga Peninsula, while the largest absolute increase was in Western Visayas, where 1.4 million people were newly covered despite a more modest relative gain of 23.3%

**Figure 5.**
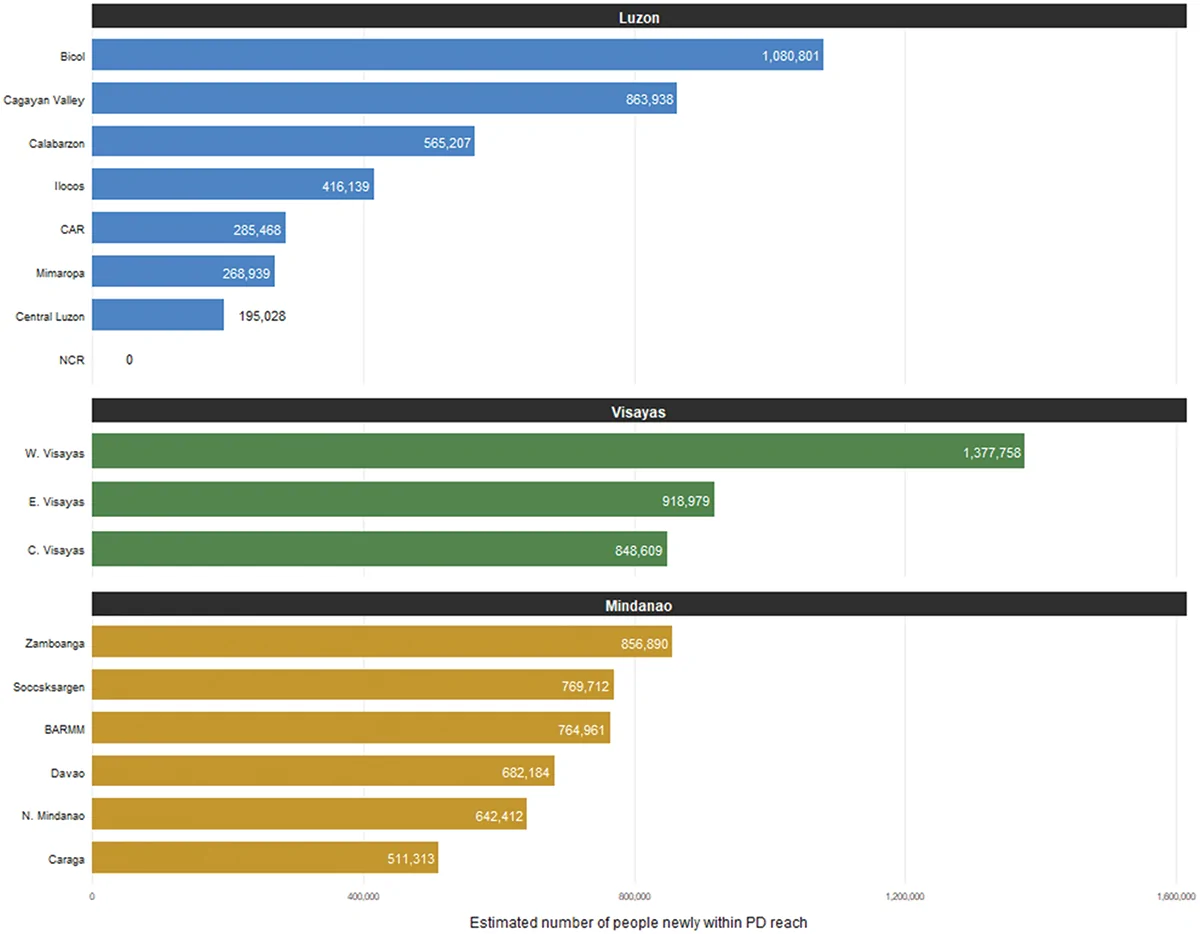
Estimated increase in population covered of PD scale-up in HD facilities.

The relative benefit of integration was less pronounced in regions that already had higher baseline access, such as Central Luzon (95.04% to 96.61%) and CALABARZON (Provinces of Cavite, Laguna, Batangas, Rizal, and Quezon) (92.34% to 95.83%), with NCR not seeing any gain as it was already at 99.74% coverage in Scenario 1. Integration also reduced disparities between regions, narrowing the gap between the highest- and lowest-access areas by around 8% points. Ownership stratification showed that because private facilities dominate numerically, most of the coverage expansion was driven by private-sector sites. Nonetheless, the inclusion of government-run facilities ensured that gains also extended to public service networks (Figure 5).

These modeled results demonstrate the potential coverage expansion achievable through PD integration, particularly in rural and underserved regions, and provide a quantitative basis for comparing equity impacts under different policy scenarios.

## Discussion

This study demonstrates that geographic inequities in dialysis access in the Philippines persist despite a decade of implementing the PD-first policy [8]. Dialysis delivery remains heavily skewed toward HD, supported by per-session reimbursement, while PD continues to lag in availability and uptake. Geospatial accessibility modelling revealed a median regional coverage of 67.9% of the population had HD access within one hour, compared to 51.3% with PD within two hours. Modeling further demonstrated that integrating PD into existing HD facilities could improve geographic coverage for an additional 11 million people who currently access HD but do not have PD as an option, with the largest relative gains in underserved rural regions. However, not all patients are clinically eligible for PD, and these estimates therefore represent potential gains in geographic availability rather than the number of patients who would ultimately received PD. These results reinforce that service integrating dialysis modalities while preserving patient choice, rather than outright selection of one modality over the other, represents a practical pathway toward reducing dialysis inequities without requiring major new infrastructure.

Placing these findings within a global context highlights both their validity and their limitations. International evidence shows that travel time is a structural determinant of dialysis continuity, with studies from India, Australia, and New Zealand reporting higher dropout rates and reduced adherence among patients living further from facilities [4,31,32]. This study confirms that such patterns are reproduced in the Philippine archipelago, where long travel distances exacerbate the burden of thrice-weekly HD. At the same time, the underutilization of PD reflects a broader global imbalance in modality distribution, despite evidence that PD offers advantages for decentralization in low- and middle-income settings and cost savings in countries with extensive programs [33,34]. In this context, decentralization denotes shifting dialysis provision from specialized hospitals, available in only a limited number of localities, to lower-level and community-based facilities, which, with targeted investment in underserved areas, may progressively broaden geographic access.

These findings extend prior research on geographic inequities in Philippine health services. The national distribution of dialysis facilities mirrors earlier studies on inpatient and outpatient access, which identified Visayas and Mindanao as particularly underserved regions [12,22]. Similar geographic disparities have been reported in admission rates, with provinces in Mindanao registering disproportionately high hospitalization burdens [35]. These patterns are consistent with long-standing structural underinvestment in rural health systems, which require patients in poorer regions to travel further to access both basic and specialized care.

A methodological contribution of this study was the adjustment of walking speeds to reflect the mobility limitations of patients with kidney failure. Using the default 5 km/h speed for healthy adults overestimated coverage, while a 3 km/h KF-adjusted speed reduced estimates by 5.0% under mixed transport. This national average, however, is not representative of regional disparities. In road-dense urban regions the difference is minimal, but it widens where motorized transport and road access are sparse, reaching 10.3% in Cagayan Valley. Thus, while this correction matters little in urban settings, it disproportionately affects the rural, geographically isolated populations. This demonstrates how conventional modeling may systematically misrepresent access, particularly when applied to chronically ill populations. While AccessMod has been widely used in maternal, child, and general health services, few studies have adapted its assumptions for NCDs. Incorporating illness-specific parameters therefore provides more realistic national estimates and highlights disparities that are otherwise hidden [29–31].

The financing environment further perpetuates the imbalance between HD and PD. HD is reimbursed on a per-session basis, incentivizing widespread adoption, while PD is limited by Z-Benefit (government financing for catastrophic health expenditure) accreditation requirements and separate claims processes [8,36]. Facilities face little incentive to integrate PD, leading to fragmented provision despite policy commitments to PD-first. This misalignment echoes broader NCD challenges where fragmented programs, weak prevention, and inconsistent implementation under the Philippine Package of Essential NCD interventions (PhilPEN) delay diagnosis and accelerate complications.

Provider attitudes alone do not account for this imbalance. A survey of Filipino nephrologists conducted in 2006 found that only 10.2% would recommend PD to an otherwise eligible patient, while nearly half deferred the choice to the patient, and that physician reimbursement ranked least important among the factors informing their recommendations. Exposure to PD during fellowship training did not predict the propensity to recommend it, leading the authors to look beyond the clinician-patient encounter for the stronger determinants of modality. They noted that very few free-standing PD units existed in the country, and argued that limited healthcare budget allotment and poor accessibility of most of the population to urban HD facilities should favor PD in a developing island nation [37]. This study finds the same scarcity two decades later, with dual-modality sites concentrated almost entirely in urban Luzon and PD accounting for only 4% of patients on kidney replacement therapy [9], which hints that the PD-first policy advanced financing without an accompanying health systems strengthening strategy, as far as could be identified, to build the service delivery, workforce, and supply capacity that PD requires. Providers cannot prescribe a modality that the system is not equipped to absorb and reliably support.

The modeling scenarios simulate the policy relevance of HD-PD integration. Scenario 4 demonstrated that embedding PD within existing HD facilities would expand geographic coverage. The geographic patterns identified in this study suggest, though they do not directly measure, that PD integration may reduce the household-level travel burden associated with hemodialysis. Patients with kidney failure routinely attend sessions accompanied by family members, so treatment location imposes financial and social costs on entire households. While these social costs were not directly modeled, the coverage gains represent the population for whom this burden could plausibly be reduced. Using the general population captures these household-level effects, linking geospatial inequities to broader patterns of social disruption.

Realizing these coverage gains depends on infrastructure beyond facility distribution. Workforce availability, including nephrologists and trained PD nurses, training capacity, and supply chain continuity for PD fluids all influence the feasibility of scaling PD services [34]. Incorporating peritoneal dialysis competencies into baseline dialysis-nurse training, delivered as a dedicated module, may reduce the training burden of integration. In the interim, the coverage gaps identified suggest that establishing LGU-level PD fluid replenishment stations also merits exploration as a logistical complement to facility-based expansion in underserved regions.

Integration should be understood, however, as a mechanism that requires deliberate implementation rather than an automatic consequence of co-locating services. In Thailand, the incorporation of PD into universal coverage advanced through sustained policy commitment and coordination across government, clinicians, and civil society rather than through infrastructure alone, and the program’s later difficulties, including falling volumes and workforce strain, indicate that such gains are not self-sustaining [38]. Comparative experience further suggests that the success of PD-favored policies is inversely related to the extent of existing hemodialysis infrastructure [39], which creates a self-perpetuating environment favoring HD use. Realizing the modeled coverage would therefore depend on enabling conditions the present analysis does not capture, including the reliance on imported PD consumables noted earlier, alongside the financing, accreditation, and workforce capacity discussed above. The estimated coverage gain represents an upper bound of what integration could achieve rather than an outcome that would follow automatically once facilities are combined.

Geography, however, is only one dimension of equity. Policymakers must not only consider availability and approachability but also acceptability, affordability, and appropriateness [14]. In the Philippine context, sustaining PD expansion requires patient and provider confidence and empowerment, adequate financing, and reliable supply chains, while addressing conflicts of interest linked to HD facility ownership may further shape recommendations. Government facilities account for only 13.7% of dialysis facilities; government incentives or regulatory mechanisms will therefore be required to engage private operators in dual-modality provision. International precedents from Hong Kong, Thailand, and Mexico demonstrate that high PD adoption is achievable through administrative integration rather than modality substitution, and that framing PD as a capacity-expanding complement to HD rather than a revenue threat improves private-sector uptake [33]. Beyond access, continuity, coordination, and responsiveness are also necessary if geographic gains are to translate into meaningful improvements in patient experience and treatment outcomes [13].

These findings argue for complementary integration of PD and HD across facilities, rather than privileging one modality over the other. Geographic modeling demonstrates that dual-modality availability would expand patient choice and reduce inequities, particularly for rural households who face the steepest barriers under the current system. Piloting PD expansion in underserved regions, supported by appropriate financing and accreditation reforms, could provide a pathway to more equitable kidney replacement therapy delivery in the Philippines.

Ultimately, dialysis access is not only a logistical concern but a test of the health system’s ability to sustain life-sustaining chronic care. Long travel distances impose financial, physical, and social costs that destabilize households and undermine patient dignity. Designing delivery models that bring care closer to patients’ homes, through PD integration and dual-modality planning, offers a pathway to protect families and reduce inequities in chronic disease management.

### Strengths and limitations

To the authors’ knowledge, this is the first national-level assessment of dialysis accessibility in the Philippines using a cost-distance analysis approach. The study demonstrates the feasibility of applying spatial accessibility analysis to chronic care services in a lower-middle-income country context. The accessibility model provides more realistic estimates of patient travel time than conventional healthy-adult assumptions by incorporating KF-adjusted walking speeds. The integration of national and provincial datasets enhances policy relevance and allows the findings to contribute to Universal Health Care implementation and service integration planning.

At the same time, several limitations are acknowledged. Modeled estimates are based on nearest facility assumptions without accounting for patient and provider preference. Facility registries were fragmented and lacked consistent information on dialysis machine counts, staffing, and operating hours, restricting the analysis to binary access estimates rather than functional capacity modeling. This gap likely led to conservative estimates of inequity, since facility throughput and local demand vary widely across settings. Travel-time assumptions, such as ideal dry-season speeds and private vehicle use, may have underestimated the actual burden for patients dependent on public or informal transport and did not take into account the additional burdens during the rainy seasons, where severe flooding and road closures are commonplace in a country in the Pacific.

Potential sources of bias were mitigated where possible. Facility lists from the Department of Health and PhilHealth were cross-checked to minimize undercounting, which helped reduce geocoding errors. Adjusting for KF walking speeds also lessened the risk of overestimating accessibility, yet bias may still persist due to incomplete accreditation data, unlisted facilities, or assumptions that all centers were fully operational. Undercounting and idealized facility functionality demonstrate the uncertainty inherent in spatial modeling using incomplete health system data.

Peritoneal dialysis scenarios captured only physical accessibility and did not account for supply chain reliability, household suitability, or clinical eligibility, which are essential for sustained PD uptake. Missing variables such as dialysis machine capacity, service hours, and workforce adequacy further influence whether geographic access translates into effective and equitable care. These data gaps highlight the need to strengthen facility-level reporting and health information systems to reflect service readiness and continuity more accurately.

While constrained by data gaps and modeling assumptions, this study demonstrates that spatial analysis can yield actionable insight into service inequities even amid imperfect information. The study establishes a transparent baseline for future work that links geographic access with clinical and policy outcomes. The findings provide a measured starting point for exploring how peritoneal dialysis integration within existing hemodialysis networks could expand equitable coverage across regions.

## Conclusions

Geographic inequity in kidney failure care persists in the Philippines despite a decade of PD-first policy, exposing a gap between national financing commitments and local service reach. In settings where health insurance expansion outpaces infrastructure development, spatial inequity becomes the next frontier of reform. Integrating PD within existing HD networks offers a geographically feasible pathway to narrow that gap without the fiscal burden of new facility construction. Sequencing that integration by current dual-modality coverage would concentrate early effort where such coverage is presently absent, in MIMAROPA and Soccsksargen at zero and BARMM at 7%, rather than distributing it uniformly across regions that are already well served. Realizing these gains would require accreditation to be restructured so that a facility already accredited for HD can add PD without entering a separate pathway and claims process. These findings can inform equity-oriented policy reforms in the Philippines and serve as a methodological template for similar analyses in other resource-limited settings.

## Supplementary Material

Supplementary_Tables_clean.docx

STROBE_Checklist_Dialysis Deserts.docx

## Data Availability

The data that support the findings of this study are available from the corresponding author, MSF, upon reasonable request.
