# Supplementary material for: Dialysis deserts in the Philippines: a national geospatial modeling study of dialysis services
Source: Glob Health Action. 2026 Sep 7;19(1):2727762. doi: 10.1080/16549716.2026.2727762 (PMC13552290; doi:10.1080/16549716.2026.2727762)
Supplement: Supplementary file 1 [file ZGHA_A_2727762_SM3981.docx]

STROBE Statement—checklist of items that should be included in reports of observational studies

|  | Item No. | Recommendation | Page  No. | Relevant text from manuscript |
| --- | --- | --- | --- | --- |
| Title and abstract | 1 | (*a*) Indicate the study’s design with a commonly used term in the title or the abstract | 1 | Abstract: Geospatial analysis was conducted using least-cost path  modeling with walking speeds adjusted for CKD functional limitations. (Line 23) |
|  |  | (*b*) Provide in the abstract an informative and balanced summary of what was done and what was found | 1 | “Objective: To assess the geographic accessibility of dialysis services and estimate potential coverage gains from integrating PD into existing hemodialysis (HD) facilities.” (lines 18–19)  “Results: In 2023, 67.9% of the population could reach an HD facility within one hour… Integrating PD into all HD sites expanded national coverage by 33.1%...” (lines 26–29) |
| Introduction | | | |  |
| Background/rationale | 2 | Explain the scientific background and rationale for the investigation being reported | 2-3 | “Dialysis access has emerged as a systemic health equity challenge… ‘dialysis deserts’ capture these inequities…” (lines 37–43)  “The Philippines ranks second in the burden of chronic kidney disease… Yet uptake has remained limited… and the system continues to be dominated by in-center HD.” (lines 51–59) |
| Objectives | 3 | State specific objectives, including any prespecified hypotheses | 3 | “The objective was to assess geographic accessibility and coverage of dialysis facilities and to estimate the potential equity gains of integrating peritoneal dialysis into existing hemodialysis networks.” (lines 98–100) |
| Methods | | | |  |
| Study design | 4 | Present key elements of study design early in the paper | 4 | “This study employed a nationwide cross-sectional geospatial design to assess dialysis facility accessibility in the Philippines.” (lines 113–116) |
| Setting | 5 | Describe the setting, locations, and relevant dates, including periods of recruitment, exposure, follow-up, and data collection | 4 | “This national analysis covers the Philippines’ 7,641 islands organized into three island groups and 18 administrative regions.” (lines 103–104)  “The country’s health service delivery operates under a devolved system… resulting in wide variation in the implementation of health programs…” (lines 105–110)  “These governance and geographic conditions made the Philippines an ideal setting to examine dialysis deserts…” (lines 108–110) |
| Participants | 6 | (*a*) *Cohort study*—Give the eligibility criteria, and the sources and methods of selection of participants. Describe methods of follow-up  *Case-control study*—Give the eligibility criteria, and the sources and methods of case ascertainment and control selection. Give the rationale for the choice of cases and controls  *Cross-sectional study*—Give the eligibility criteria, and the sources and methods of selection of participants | 5 | Although no human participants were sampled, the manuscript clearly defines the inclusion criteria for facilities and population data used in the analysis:  “All dialysis facilities accredited as of 2023 were included in the analysis. Facility data were obtained from the Department of Health Facility Registry and the PhilHealth Dialysis Facility Registry… The registries were manually harmonized, cleaned, and cross-validated to remove duplicates and closed sites, resulting in a total of 903 operational facilities nationwide.” (lines 123–130)  Population selection is defined as:  “Population data were drawn from the 2020 national census… Gridded population data… were used to generate a 100-meter raster representing population density and distribution across the country.” (lines 135–139) |
|  |  | (*b*) *Cohort study*—For matched studies, give matching criteria and number of exposed and unexposed  *Case-control study*—For matched studies, give matching criteria and the number of controls per case |  | NA |
| Variables | 7 | Clearly define all outcomes, exposures, predictors, potential confounders, and effect modifiers. Give diagnostic criteria, if applicable | 4-7 | Primary outcome defined as geographic accessibility, expressed as coverage within time thresholds:  “Travel-time coverage was used as a proxy for geographic access…” (lines 114–115)  “Accessibility thresholds… were set at one hour for HD and two hours for PD.” (lines 182–187)  Predictor and exposure variables include: • Facility characteristics: “Facilities were classified by ownership (government or private), facility type (freestanding or hospital-based), and dialysis modality…” (lines 131–134) • Spatial determinants: “Spatial datasets representing travel-time determinants included a digital elevation model (DEM), land cover, hydrography, and the road network.” (lines 142–147) • Walking speed adjustments representing functional limitations: “The study recognizes that people with CKD would have slower walking speeds… a walking speed of 3 km/h was applied rather than the conventional 5 km/h…” (lines 210–214) |
| Data sources/ measurement | 8* | For each variable of interest, give sources of data and details of methods of assessment (measurement). Describe comparability of assessment methods if there is more than one group | 5-7 | “Facility data were obtained from the Department of Health Facility Registry and the PhilHealth Dialysis Facility Registry… addresses were geocoded through the Google Maps API using R and validated against administrative boundaries and OpenStreetMap reference layers.” (lines 123–130)  Ancillary spatial datasets and measurement methods: “The DEM was sourced from the AW3D30 (JAXA) dataset… Land cover data were obtained from the 2018 Philippine GIS Data Clearinghouse… Hydrographic features… were considered impassable except where intersected by road bridges. The 2025 OpenStreetMap road network… was cleaned and categorized into Motorway, Primary, Secondary…” (lines 142–153)  Comparability ensured through harmonization: “All spatial layers were harmonized to a single projection… Raster layers were resampled to a uniform resolution of 100m x 100m.” (lines 158–161)  Travel-time modeling assessment details: “The slope derived from the DEM was used to adjust walking speeds based on Tobler’s formula… Land cover types… were assigned different walking speeds… road networked were given motorized vehicle speeds.” (lines 200–209) |
| Bias | 9 | Describe any efforts to address potential sources of bias | 16-17 | Bias from healthy vs CKD mobility assumptions mitigated: “Adjusting for CKD walking speeds also lessened the risk of overestimating accessibility…” (lines 430–432)  Limitations acknowledging residual bias: “Facility registries were fragmented and lacked consistent information on dialysis machine counts, staffing, and operating hours… Travel-time assumptions… may have underestimated the actual burden…” (lines 421–427) |
| Study size | 10 | Explain how the study size was arrived at | 5 | “All dialysis facilities accredited as of 2023 were included… resulting in a total of 903 operational facilities nationwide. No sampling or imputation was applied.” (lines 123–134)  Population dataset fully inclusive: “Gridded population data… were used to generate a 100-meter raster representing population density…” (lines 135–139)  Because this is a national cross-sectional geospatial study, the “study size” reflects the universe of facilities and the full national population, not a sample. |

| Quantitative variables | 11 | Explain how quantitative variables were handled in the analyses. If applicable, describe which groupings were chosen and why | 4-7 | Travel-time and coverage values treated as continuous rasters:  “For each scenario, travel-time models were generated and overlaid with the 2020 population grid to compute the share of the population within the defined thresholds.” (lines 236–237)  “Accessibility thresholds… were set at one hour for HD and two hours for PD.” (lines 182–187)  Walking speed is treated as continuous variable but modeled under two scenarios: healthy vs CKD-adjusted: “Coverage estimates generated under the standard healthy adult walking speed (5 km/h) were compared with those based on a CKD-adjusted speed (3 km/h).” (lines 226–229) |
| --- | --- | --- | --- | --- |
| Statistical methods | 12 | (*a*) Describe all statistical methods, including those used to control for confounding | 6-7 | The study uses geospatial modeling.  “Travel-time models were calculated using the ‘Merge Land Cover’ and ‘Accessibility’ functions within the AccessMod software.” (lines 196–203)  Road speeds and friction values assigned systematically: “Land cover types… were assigned different walking speeds, while road networks were given motorized vehicle speeds.” (lines 207–209) |
|  |  | (*b*) Describe any methods used to examine subgroups and interactions | 5-12 | Subgroup analyses performed by region and ownership: “Facility ownership (public or private), type… and administrative region were used as descriptive stratifiers…” (lines 190–191) Coverage outputs reported for each region in Figure 4 (lines 293–307). |
|  |  | (*c*) Explain how missing data were addressed | 16 | Missingness in facility datasets discussed:  “Facility registries were fragmented and lacked consistent information on dialysis machine counts, staffing, and operating hours…” (lines 421–423)  Missing facility coordinates were resolved:  “Addresses were geocoded… and validated against administrative boundaries… Coordinates outside the correct province or region were manually corrected.” (lines 127–130)  No imputation was applied (line 133). |
|  |  | (*d*) *Cohort study*—If applicable, explain how loss to follow-up was addressed  *Case-control study*—If applicable, explain how matching of cases and controls was addressed  *Cross-sectional study*—If applicable, describe analytical methods taking account of sampling strategy | NA | Not applicable because the study analyzes a full census of facilities and population rasters with no sampling. |
|  |  | (*e*) Describe any sensitivity analyses | 9 | The manuscript presents two forms of sensitivity analyses. First, the primary analysis used a CKD-adjusted walking speed of 3 km/h to represent mobility limitations among dialysis patients. A secondary analysis applied the conventional 5 km/h healthy-adult assumption to test how sensitive the travel-time estimates were to uncertainty in mobility parameters. This comparison allowed assessment of the degree to which accessibility coverage is affected by variation in plausible walking speeds.  Second, motorized travel speeds were varied by ±20% across all road classes to reflect uncertainty due to terrain, traffic, and seasonal conditions. These robustness checks are described in:  “Coverage estimates generated under the standard healthy adult walking speed (5 km/h) were compared with those based on a CKD-adjusted speed (3 km/h)… motorized travel speeds were varied by ±20% across all road classes…” (lines 226–232) |
| Results | | | | |
| Participants | 13* | (a) Report numbers of individuals at each stage of study—eg numbers potentially eligible, examined for eligibility, confirmed eligible, included in the study, completing follow-up, and analysed | 8 | “All dialysis facilities accredited as of 2023 were included… resulting in a total of 903 operational facilities nationwide.” (lines 123–130) Counts broken down in Table 1: “HD Only = 851; PD Only = 9; HD+PD = 43; Total = 903.” (lines 244–249) |
|  |  | (b) Give reasons for non-participation at each stage | NA | Not applicable because no participants were recruited, no exclusions beyond invalid/duplicate facility records. |
|  |  | (c) Consider use of a flow diagram | NA | NA |
| Descriptive data | 14* | (a) Give characteristics of study participants (eg demographic, clinical, social) and information on exposures and potential confounders | 8 | Facility characteristics summarized descriptively:  “In 2023, a total of 903 dialysis facilities were accredited nationwide… The majority (86.3%) were privately owned… 65% were freestanding clinics… HD is still the dominant modality…” (lines 244–249)  Population characteristics included by inclusion of PSA census rasters (lines 135–139). |
|  |  | (b) Indicate number of participants with missing data for each variable of interest | 16 | Coordinates missing for some facilities but corrected manually: (lines 127–130). |
|  |  | (c) *Cohort study*—Summarise follow-up time (eg, average and total amount) | NA | NA |
| Outcome data | 15* | *Cohort study*—Report numbers of outcome events or summary measures over time | NA | NA |
|  |  | *Case-control study—*Report numbers in each exposure category, or summary measures of exposure | NA | NA |
|  |  | *Cross-sectional study—*Report numbers of outcome events or summary measures | 10-12 | Primary outcome = population coverage within travel-time thresholds.  Examples:  “Nationally, 67.9%… could reach an HD facility within one hour, while only 51.3% could reach a PD facility within two hours.” (lines 293–297)  Region-level outcomes displayed in Figures 4 and 5 (pages 11–13). |
| Main results | 16 | (*a*) Give unadjusted estimates and, if applicable, confounder-adjusted estimates and their precision (eg, 95% confidence interval). Make clear which confounders were adjusted for and why they were included | 9-13 | This study uses deterministic cost-distance modeling, in which accessibility outcomes are computed directly from spatial inputs and travel-speed parameters. Because the analysis does not rely on sampling or statistical estimation, the outputs represent fixed model results rather than inferential estimates. The primary outcomes which was set as the population coverage within 1-hour (HD) and 2-hour (PD) travel-time thresholds, are presented as absolute percentages at national and regional levels. These estimates represent the modeled share of the population able to reach a dialysis facility within the defined thresholds under each scenario.  For example, “Using the conventional healthy adult speed of 5 km/h, national coverage was 74.9% for HD… When the walking speed was adjusted to 3 km/h… coverage dropped to 68.0% for HD and 51.3% for PD.” (lines 269–275)  Because the analysis does not use regression or confounder adjustment, precision is shown through scenario and sensitivity analyses, including alternative walking-speed assumptions and ±20% variation in motorized travel speeds, which demonstrate robustness rather than confidence intervals. |
|  |  | (*b*) Report category boundaries when continuous variables were categorized | 6, 8 | Time thresholds (categories) reported:  “1-hour for HD and 2-hours for PD.” (lines 182–187)  Facility ownership categories defined in Table 1 (lines 244–249). |
|  |  | (*c*) If relevant, consider translating estimates of relative risk into absolute risk for a meaningful time period | NA | NA |

Continued on next page

| Other analyses | 17 | Report other analyses done—eg analyses of subgroups and interactions, and sensitivity analyses | 9-13 | Subgroup analyses examined geographic accessibility by administrative region and by facility ownership, illustrating structural differences in service availability (lines 304–306; 320–323).  Sensitivity analyses tested the robustness of modeled estimates by varying key parameters: walking-speed assumptions (3 km/h CKD-adjusted vs 5 km/h healthy adult) and ±20% variation in motorized travel speeds across road classes (lines 226-232). |
| --- | --- | --- | --- | --- |
| Discussion | | | | |
| Key results | 18 | Summarise key results with reference to study objectives | 14 | “This study demonstrates that geographic inequities in dialysis access in the Philippines persist despite a decade of implementing the PD-first policy.” (lines 334–335)  “Geospatial accessibility modelling revealed that 67.9% of the population had HD access within one hour, compared to 51.3% with PD…” (lines 338–339)  “Modeling further demonstrated that integrating PD into existing HD facilities could improve geographic coverage for an additional 24 million people…” (lines 340–342) |
| Limitations | 19 | Discuss limitations of the study, taking into account sources of potential bias or imprecision. Discuss both direction and magnitude of any potential bias | 16–17 | “Modeled estimates are based on nearest facility assumptions without accounting for patient and provider preference.” (lines 420–421)  “Facility registries were fragmented and lacked consistent information on dialysis machine counts, staffing, and operating hours… restricting the analysis to binary access estimates…” (lines 421–424)  “Travel-time assumptions… may have underestimated the actual burden for patients dependent on public or informal transport.” (lines 426–427)  “Bias may still persist due to incomplete accreditation data, unlisted facilities, or assumptions that all centers were fully operational.” (lines 431–432) |
| Interpretation | 20 | Give a cautious overall interpretation of results considering objectives, limitations, multiplicity of analyses, results from similar studies, and other relevant evidence | 14-16 | “These results reinforce that service integration… represents a practical pathway toward reducing dialysis inequities without requiring major new infrastructure.” (lines 342–344)  “A methodological contribution of this study was the adjustment of walking speeds… Using the default 5 km/h speed overstated coverage… CKD-adjusted speed reduced estimates…” (lines 365–368)  “Geography, however, is only one dimension of equity… sustaining PD expansion requires patient and provider confidence, adequate financing, and reliable supply chains…” (lines 389–392)  “These considerations highlight why improving geographic access must be accompanied by broader system reforms.” (lines 396–397) |
| Generalisability | 21 | Discuss the generalisability (external validity) of the study results | 15–16 | “These findings extend prior research on geographic inequities in Philippine health services… patterns consistent with long-standing structural underinvestment in rural health systems.” (lines 357–364)  “While this study focused on the Philippines, its lessons resonate with broader global debates on chronic care access.” (lines 460–461)  “In other settings, home-manageable modalities like PD have been leveraged to decentralize kidney care and reduce the geographic divide.” (lines 461–462) |
| Other information | |  | | |
| Funding | 22 | Give the source of funding and the role of the funders for the present study and, if applicable, for the original study on which the present article is based | 17 | “The study was supported by the Belgian Directorate-General for Development Cooperation and Humanitarian Aid (DGD), which had no role in the study design, data interpretation, or decision to publish.” (lines 466–471) |

*Give information separately for cases and controls in case-control studies and, if applicable, for exposed and unexposed groups in cohort and cross-sectional studies.

Note: An Explanation and Elaboration article discusses each checklist item and gives methodological background and published examples of transparent reporting. The STROBE checklist is best used in conjunction with this article (freely available on the Web sites of PLoS Medicine at http://www.plosmedicine.org/, Annals of Internal Medicine at http://www.annals.org/, and Epidemiology at http://www.epidem.com/). Information on the STROBE Initiative is available at www.strobe-statement.org.
