# Supplementary material for: Dialysis deserts in the Philippines: a national geospatial modeling study of dialysis services
Source: Glob Health Action. 2026 Sep 7;19(1):2727762. doi: 10.1080/16549716.2026.2727762 (PMC13552290; doi:10.1080/16549716.2026.2727762)
Supplement: Supplementary file 2 [file ZGHA_A_2727762_SM3982.docx]

## Annex 1: Spatial Data Inputs and Preparation

| **Data type** | **Source / Year** | **Description** | **Processing steps** |
| --- | --- | --- | --- |
| Administrative boundaries | PSA, NAMRIA (2020) | National–regional–provincial shapefiles | Harmonized to EPSG:32651 (UTM Zone 51N) |
| Road network | OpenStreetMap (2023) | National road and transport network | Reclassified by road type |
| Rivers & waterways | NAMRIA hydrographic data | Major rivers, lakes, and coastlines | Treated as barriers except at defined crossings |
| Dialysis facilities | PhilHealth registry (2023); DOH list | HD and PD facility names and coordinates | Cleaned in R, geocoded using Google Maps API, and validated manually |
| Population density | WorldPop / PSA Census (2020) | 100m gridded population estimates | Resampled and aligned to the analysis grid |
| Elevation | JAXA DEM (latest release) | 30m resolution Digital Elevation Model | Resampled to 100m; harmonized projection |
| Land cover | NAMRIA (2020) | Land cover classification | Reclassified into travel categories (urban, forest, agriculture, water, etc.) |

## Annex 2: AccessMod Methodology and Parameters

| Component | Description | Parameter / Setting | Notes |
| --- | --- | --- | --- |
| Software and version | WHO AccessMod 5 (v5.8) | Offline desktop application | Same version used for all scenarios |
| Coordinate system | WGS 84 / UTM Zone 51N (EPSG:32651) | Unified for all inputs | Ensures alignment of raster layers |
| Analysis extent | National coverage | 100m grid resolution | Matches population raster resolution |
| Accessibility module | Cost-distance analysis | Least-cost path algorithm | Models travel time from population to nearest facility |
| Travel modes and thresholds | Motorized, walking, mixed | 1-hour and 2-hour thresholds | Benchmarks for coverage analysis |
| Walking speeds (healthy) | 5 km/h | Default assumption | WHO rural standard |
| Walking speeds (KF-adjusted) | 3 km/h | Reduced mobility scenario | Reflects KF-related limitations |
| Dry-season travel speeds | | | |
| – Paved road | 60 km/h |  | National highway speed |
| – Secondary road | 40 km/h |  | Provincial/municipal roads |
| – Tertiary/unpaved road | 20 km/h |  | Rural access roads |
| – Urban/built-up | 15 km/h |  | Adjusted for traffic |
| – Grassland/agriculture | 5 km/h |  | Moderate terrain |
| – Forest | 2 km/h |  | Difficult terrain |
| – Water bodies | Barrier |  | Except where crossings defined |
| Scenarios modeled | Healthy vs KF walking speeds  1. HD-only  2. PD-only  3. HD+PD network  4. PD scale-up | Comparative scenarios | Evaluated accessibility coverage by region |
| Output metrics | Travel-time rasters, population coverage | Summary per scenario | Used for policy interpretation |

## Annex 3: Supplementary Results — Facility and Accessibility Summary

| Indicator | Definition | Measure | Source / Notes |
| --- | --- | --- | --- |
| Total facilities (N) | Licensed dialysis facilities included in AccessMod analysis | N = 903  total: HD Only = 851 (94.2%), PD Only = 9 (1.0%), HD + PD = 43 (4.8%) | PhilHealth registry (2023); DOH list |
| Ownership | Public vs private ownership | Government = 124 (13.7%); Private = 779 (86.3%) | DOH Facility Masterlist 2023 |
| Facility type | Freestanding vs hospital-based facilities | Freestanding = 65%; Hospital-based = 35% | Derived from DOH accreditation records |
| Regional distribution | Regional count of dialysis facilities | Highest: NCR (190); Lowest: BARMM (5) | DOH registry and PhilHealth records |
| Dual-modality coverage | Facilities accredited for both HD and PD | 43 facilities (4.8% of total);  24 (55.8%) government-run | DOH accreditation list 2023 |

## Annex 4: Area-based and Population-based Densities, By Region

| Regions | per 1000km2 | per 1Mpop |
| --- | --- | --- |
| Region I (Ilocos Region) | 3.66 | 8.49 |
| Region II (Cagayan Valley) | 1.06 | 7.60 |
| Region III (Central Luzon) | 7.56 | 12.96 |
| Region IV-A (Calabarzon) | 8.08 | 7.90 |
| Region V (Bicol Region) | 2.77 | 7.89 |
| Region VI (Western Visayas) | 2.84 | 7.17 |
| Region VII (Central Visayas) | 3.36 | 5.94 |
| Region VIII (Eastern Visayas) | 1.25 | 5.72 |
| Region IX (Zamboanga Peninsula) | 0.55 | 2.06 |
| Region X (Northern Mindanao) | 2.63 | 9.16 |
| Region XI (Davao Region) | 1.54 | 5.53 |
| Region XII (Soccsksargen) | 1.44 | 5.73 |
| National Capital Region (NCR) | 317.44 | 14.09 |
| Cordillera Administrative Region (CAR) | 1.76 | 17.80 |
| Region XIII (Caraga) | 1.01 | 6.77 |
| Region IV-B (Mimaropa) | 0.30 | 2.48 |
| Bangsamoro Autonomous Region In Muslim Mindanao (BARMM) | 0.37 | 1.01 |
|  |  |  |
| National | 1.76 | 7.17 |

## Annex 5: Percentage of Covered Populations under healthy compared to KF walking speeds

|  | Walking Only | |  | Walking + Motorized | |  |
| --- | --- | --- | --- | --- | --- | --- |
| Region | Healthy | Kidney Failure | **Rel. Diff** | Healthy | Kidney Failure | **Rel. Diff** |
| Region I (Ilocos Region) | 31.8 (25.2 - 38.3) | 18.7 (15.1 - 22.7) | 41.1% | 92.5 (87.2 - 94.9) | 88.7 (71.2 - 90.2) | 4.1% |
| Region II (Cagayan Valley) | 19.4 (16.5 - 22.2) | 13.2 (10.9 - 15.2) | 32.1% | 74.9 (63.4 - 81.4) | 67.2 (44.6 - 70.1) | 10.3% |
| Region III (Central Luzon) | 56.0 (47.8 - 63.4) | 38.4 (31.6 - 44.2) | 31.5% | 95.8 (94.9 - 96.2) | 95.0 (85.5 - 95.4) | 0.8% |
| Region IV-A (Calabarzon) | 68.6 (63.0 - 73.2) | 54.7 (47.0 - 60.1) | 20.3% | 93.3 (91.9 - 94.4) | 92.3 (86.7 - 92.7) | 1.0% |
| Region V (Bicol Region) | 22.7 (19.3 - 26.3) | 15.8 (13.5 - 18.0) | 30.4% | 71.1 (65.1 - 76.1) | 67.2 (49.4 - 68.6) | 5.5% |
| Region VI (Western Visayas) | 24.2 (21.4 - 26.7) | 18.7 (16.7 - 20.4) | 22.5% | 78.0 (70.2 - 83.8) | 74.4 (58.5 - 75.7) | 4.7% |
| Region VII (Central Visayas) | 41.3 (36.4 - 45.6) | 30.4 (25.5 - 34.2) | 26.4% | 81.8 (77.5 - 84.6) | 79.4 (67.8 - 80.3) | 2.9% |
| Region VIII (Eastern Visayas) | 17.5 (15.2 - 19.8) | 12.5 (10.8 - 14.2) | 28.6% | 66.8 (58.2 - 72.5) | 61.1 (41.6 - 63.2) | 8.6% |
| Region IX (Zamboanga Peninsula) | 17.6 (13.9 - 20.3) | 10.2 (8.1 - 12.4) | 42.0% | 46.5 (41.7 - 50.7) | 44.0 (35.0 - 44.9) | 5.6% |
| Region X (Northern Mindanao) | 33.0 (29.5 - 36.0) | 25.2 (22.2 - 27.9) | 23.8% | 86.5 (80.3 - 90.0) | 82.6 (63.3 - 84.1) | 4.5% |
| Region XI (Davao Region) | 33.6 (31.1 - 35.5) | 27.2 (24.0 - 29.7) | 19.1% | 70.4 (65.6 - 74.1) | 67.8 (56.1 - 68.7) | 3.7% |
| Region XII (Soccsksargen) | 25.3 (21.6 - 28.6) | 17.5 (14.8 - 20.1) | 30.7% | 72.3 (66.7 - 76.4) | 69.0 (53.4 - 70.3) | 4.5% |
| National Capital Region (NCR) | 99.7 (99.5 - 99.7) | 98.0 (93.9 - 99.3) | 1.7% | 99.7 (99.7 - 99.7) | 99.7 (99.7 - 99.7) | 0.0% |
| Cordillera Administrative Region (CAR) | 36.8 (33.1 - 40.3) | 29.2 (26.2 - 31.5) | 20.7% | 73.2 (67.6 - 77.5) | 67.9 (52.6 - 69.8) | 7.2% |
| Region XIII (Caraga) | 21.5 (18.7 - 23.6) | 14.9 (12.0 - 17.5) | 30.7% | 68.1 (58.5 - 74.2) | 62.4 (42.2 - 64.6) | 8.4% |
| Mimaropa Region | 11.2 (9.8 - 12.5) | 8.0 (6.7 - 9.0) | 29.1% | 37.9 (34.7 - 39.7) | 36.0 (27.0 - 36.7) | 4.8% |
| Bangsamoro Autonomous Region In Muslim Mindanao (BARMM) | 19.6 (18.5 - 20.6) | 16.5 (14.9 - 17.8) | 15.9% | 54.9 (48.6 - 59.9) | 50.6 (36.5 - 52.1) | 7.9% |

## Annex 5: Percentage of Covered Populations under different scenarios

| Region |  | HD Coverage  (1hDFA) | PD Coverage (2hDFA) | Current HD+PD  (2hDFA) | All HD with PD  (2hDFA) |
| --- | --- | --- | --- | --- | --- |
| Ilocos |  | 88.68 (83.63–92.61) | 85.54 (72.87–85.54) | 85.54 (95.86–88.52) | 96.53 (95.86–96.75) |
| Cagayan Valley |  | 67.21 (56.67–74.06) | 46.48 (33.42–46.48) | 46.48 (84.40–53.63) | 90.65 (84.40–92.90) |
| C. Luzon |  | 95.04 (93.73–95.55) | 91.93 (89.66–91.93) | 91.93 (96.28–93.65) | 96.61 (96.28–96.81) |
| Calabarzon |  | 92.34 (90.78–93.18) | 91.39 (89.14–91.39) | 91.35 (95.06–92.63) | 95.83 (95.06–96.31) |
| Bicol |  | 67.17 (60.52–70.54) | 66.30 (60.58–66.30) | 65.63 (79.79–70.90) | 84.94 (79.79–86.81) |
| W. Visayas |  | 74.37 (66.52–78.91) | 67.59 (56.69–67.59) | 67.59 (88.76–72.65) | 91.69 (88.76–93.22) |
| C. Visayas |  | 79.41 (74.94–81.23) | 51.88 (48.06–51.88) | 51.88 (86.73–54.58) | 89.91 (86.73–91.38) |
| E. Visayas |  | 61.08 (52.64–64.56) | 33.33 (28.30–33.33) | 33.33 (76.11–37.32) | 81.29 (76.11–83.60) |
| Zamboanga |  | 43.95 (39.42–46.99) | 21.74 (20.89–21.74) | 21.74 (55.31–22.42) | 66.06 (55.31–71.86) |
| N. Mindanao |  | 82.55 (75.83–85.60) | 46.84 (38.41–46.84) | 46.24 (92.54–51.09) | 95.34 (92.54–96.24) |
| Davao |  | 67.76 (62.88–70.73) | 67.60 (62.07–67.60) | 67.60 (76.70–70.62) | 80.77 (76.70–83.41) |
| Soccsksargen |  | 69.02 (63.20–72.44) | 38.86 (23.63–38.86) | 0.00 (80.86–0.00) | 86.67 (80.86–90.22) |
| NCR |  | 99.74 (99.74–99.74) | 99.74 (99.74–99.74) | 99.74 (99.74–99.74) | 99.74 (99.74–99.74) |
| CAR |  | 67.92 (62.83–70.54) | 49.38 (39.61–49.38) | 49.38 (78.46–59.29) | 83.80 (78.46–85.99) |
| Caraga |  | 62.37 (52.65–67.75) | 43.21 (35.61–43.21) | 43.21 (76.61–50.80) | 80.60 (76.61–82.48) |
| Mimaropa |  | 36.04 (32.87–37.53) | 0.00 (0.00–0.00) | 0.00 (41.41–0.00) | 44.37 (41.41–47.60) |
| BARMM |  | 50.57 (44.74–54.42) | 51.31 (34.10–51.31) | 7.21 (62.20–12.53) | 66.04 (62.20–67.80) |
| Region I: Ilocos Region, Region II: Cagayan Valley, Region III: Central Luzon, Region IV-A: Calabarzon, Region V: Bicol Region, Region VI: Western Visayas, Region VII: Central Visayas, Region VIII: Eastern Visayas, Region IX: Zamboanga Peninsula, Region X: Northern Mindanao, Region XI: Davao Region, Region XII: Soccsksargen, NCR: National Capital Region, CAR: Cordillera Administrative Region, Region XIII: Caraga, Mimaropa: Mindoro-Marinduque-Romblon-Palawan, BARMM: Bangsamoro Autonomous Region in Muslim Mindanao | | | | | |

## Annex 6: Estimated additional population covered under PD integration scenario

| **Region** | **Population (2020 PSA)** | **HD Only  (%)** | **PD Integration (%)** | **Gain vs HD (pp)** | **Gain vs HD (Rel %)** | **Newly Covered vs HD** |
| --- | --- | --- | --- | --- | --- | --- |
| **Ilocos Region** | 5,301,139 | 88.68 | 96.53 | 7.85 | 8.9% | 416,139 |
| **Cagayan Valley** | 3,685,744 | 67.21 | 90.65 | 23.44 | 34.9% | 863,938 |
| **Central Luzon** | 12,422,172 | 95.04 | 96.61 | 1.57 | 1.7% | 195,028 |
| **Calabarzon** | 16,195,042 | 92.34 | 95.83 | 3.49 | 3.8% | 565,207 |
| **Bicol Region** | 6,082,165 | 67.17 | 84.94 | 17.77 | 26.5% | 1,080,801 |
| **NCR** | 13,484,462 | 99.74 | 99.74 | 0 | 0.0% | - |
| **CAR** | 1,797,660 | 67.92 | 83.8 | 15.88 | 23.4% | 285,468 |
| **Mimaropa** | 3,228,558 | 36.04 | 44.37 | 8.33 | 23.1% | 268,939 |
| **Western Visayas** | 7,954,723 | 74.37 | 91.69 | 17.32 | 23.3% | 1,377,758 |
| **Central Visayas** | 8,081,988 | 79.41 | 89.91 | 10.5 | 13.2% | 848,609 |
| **Eastern Visayas** | 4,547,150 | 61.08 | 81.29 | 20.21 | 33.1% | 918,979 |
| **Zamboanga Peninsula** | 3,875,576 | 43.95 | 66.06 | 22.11 | 50.3% | 856,890 |
| **Northern Mindanao** | 5,022,768 | 82.55 | 95.34 | 12.79 | 15.5% | 642,412 |
| **Davao Region** | 5,243,536 | 67.76 | 80.77 | 13.01 | 19.2% | 682,184 |
| **Soccsksargen** | 4,360,974 | 69.02 | 86.67 | 17.65 | 25.6% | 769,712 |
| **Caraga** | 2,804,788 | 62.37 | 80.6 | 18.23 | 29.2% | 511,313 |
| **BARMM** | 4,944,800 | 50.57 | 66.04 | 15.47 | 30.6% | 764,961 |
| GRAND TOTAL | 109,033,245 |  |  |  |  | 11,048,338 |
